# Novel *TMEM98, MFRP, PRSS56* variants in a large United States high hyperopia and nanophthalmos cohort

**DOI:** 10.1038/s41598-020-76725-8

**Published:** 2020-11-17

**Authors:** Lev Prasov, Bin Guan, Ehsan Ullah, Steven M. Archer, Bernadete M. Ayres, Cagri G. Besirli, Laurel Wiinikka-Buesser, Grant M. Comer, Monte A. Del Monte, Susan G. Elner, Sarah J. Garnai, Laryssa A. Huryn, Kayla Johnson, Shivani S. Kamat, Philip Lieu, Shahzad I. Mian, Christine A. Rygiel, Jasmine Y. Serpen, Hemant S. Pawar, Brian P. Brooks, Sayoko E. Moroi, Julia E. Richards, Robert B. Hufnagel

**Affiliations:** 1 Department of Ophthalmology and Visual Sciences, University of Michigan, Ann Arbor, MI 48105 USA; 2 Department of Human Genetics, University of Michigan, Ann Arbor, MI 48109 USA; 3 Ophthalmic Genetics and Visual Function Branch, National Eye Institute, National Institutes of Health, Bethesda, MD 20892 USA; 4 Department of Ophthalmology and Visual Sciences, The Ohio State University, Columbus, OH 43212 USA; 5 Case Western Reserve University School of Medicine, Cleveland, OH 44106 USA

**Keywords:** Visual system, Molecular medicine, Genetics, Disease genetics, Genetic testing

## Abstract

Nanophthalmos is a rare condition defined by a small, structurally normal eye with resultant high hyperopia. While six genes have been implicated in this hereditary condition (*MFRP, PRSS56, MYRF, TMEM98, CRB1,VMD2/BEST1*), the relative contribution of these to nanophthalmos or to less severe high hyperopia (≥ + 5.50 spherical equivalent) has not been fully elucidated. We collected probands and families (n = 56) with high hyperopia or nanophthalmos (≤ 21.0 mm axial length). Of 53 families that passed quality control, plausible genetic diagnoses were identified in 10/53 (18.8%) by high-throughput panel or pooled exome sequencing. These include 1 *TMEM98* family (1.9%), 5 *MFRP* families (9.4%), and 4 *PRSS56* families (7.5%), with 4 additional families having single allelic hits in *MFRP* or *PRSS56* (7.5%). A novel deleterious *TMEM98* variant (NM_015544.3, c.602G>C, p.(Arg201Pro)) segregated with disease in 4 affected members of a family. Multiple novel missense and frameshift variants in *MFRP* and *PRSS56* were identified. *PRSS56* families were more likely to have choroidal folds than other solved families, while *MFRP* families were more likely to have retinal degeneration. Together, this study defines the prevalence of nanophthalmos gene variants in high hyperopia and nanophthalmos and indicates that a large fraction of cases remain outside of single gene coding sequences.

## Introduction

Uncorrected refractive error is a leading cause of visual impairment in the United States and worldwide^1,2^. Hyperopia or farsightedness is the result of small eye size (short axial length) or flat corneal diameter, and is a highly heritable trait, estimated 70–90% from twin studies^3,4^. The extreme of this condition is nanophthalmos, which is characterized by a small, but structurally intact eye. This condition has been variably defined in the literature based on features of axial length, scleral wall thickness, and anterior segment dimensions^5^. Nanophthalmos can be a significant burden on vision, leading to angle closure glaucoma, uveal effusion, retinal detachment, complications with cataract surgery, strabismus and amblyopia^6–13^. To date, the molecular pathogenesis of this condition remains elusive, but it is thought to be a disorder of normal eye growth, though distinct from microphthalmia which arises from gross ocular malformations^5^.

Six genes (*MFRP, PRSS56, MYRF, TMEM98, CRB1,VMD2/BEST1*) have been implicated in this condition^5,14–19^, with two causing distinctive retinal phenotypes leading to different clinical classification: *CRB1* is associated with Leber congenital amaurosis, early onset retinal dystrophy, retinitis pigmentosa, and maculopathies^20,21^. *BEST1* is associated with autosomal dominant vitreoretinochoroidopathy, vitelliform macular dystrophy, and autosomal-recessive bestrophinopathy^22^. Genome-wide association studies of myopia and hyperopia have also implicated both *PRSS56* and *TMEM98* in refraction disorders^23^ suggesting that these genes may modulate less extreme forms of hyperopia. There are conflicting estimates regarding the fraction of extreme hyperopia and nanophthalmos cases explained by variants in known genes, and these are likely affected by inclusion criteria for particular studies and the sampled populations^24,25^.

We previously collected a large cohort of nanophthalmos and high hyperopia families with sporadic, autosomal dominant, and autosomal recessive inheritance^14^. We found that *MYRF* coding variants explain a very small portion of these families^14^. Here, we evaluate the burden of pathogenic variants in the known nanophthalmos genes (*MFRP*, *PRSS56*, *MYRF*, *TMEM98*) among an expanded cohort of United States families, using a combination of linkage analysis and pooled exome sequencing, and a high-throughput sequencing panel, and we explore the clinical features among these genotypic classes.

## Materials and methods

### Human subjects and clinical testing

This study was performed under protocols approved by the Insitutional Review Boards of the University of Michigan and the Office of Human Research Subject Protection at the National Institutes of Health, in accordance with the Common Rule of the United States Federal Government (46CFR45). All subjects provided written informed consent. Subjects were evaluated predominantly at two clinical sites: the University of Michigan Kellogg Eye Center and the Ophthalmic Genetics and Visual Function Branch Clinic at the National Eye Institute. Most of the subjects included for this study were from a previously collected cohort screened for *MYRF* coding variants^14^ and clinical features of 8 of these were described in a case series^26^. When available, clinical records were reviewed from additional enrolled family members offsite, and blood samples were collected for DNA extraction. Individuals self-identified race was recorded. When possible, patients had standard ophthalmic clinical evaluation, including best corrected visual acuity (BCVA), refraction, tonometry, gonioscopy, slit lamp biomicroscopy, and fundoscopy. When clinically indicated or appropriate, patients also underwent fundus color and autofluorescence imaging (Topcon, Tokyo, Japan; Optos, Dunfermline, Scotland), optical coherence tomography (Cirrus HD-OCT, Carl Zeiss Meditec, Dublin, CA; Spectralis, Heidelberg, Germany), B-scan and ultrasound biomicroscopy (UBM) performed with the Aviso S (Quantel Medical, Cournon-d’Auvergne, France) or Eye Cubed instruments (Ellex, Adelaide, Australia), and optical biometry (IOLMaster, Carl Zeiss Meditec; Lenstar, Haag-Streit, Köniz, Switzerland). Clinical criteria for inclusion for the nanophthalmos phenotype included axial length ≤ 21.0 mm in the more affected eye, with a difference of less than 2.0 mm between the two eyes. High hyperopes were included based on a phakic cycloplegic or manifest refraction of ≥  + 5.50 spherical equivalents (SE) in at least one eye with no more than 3 diopters of anisometropia. Presence of coloboma or gross ocular malformation was an exclusion criterion.

### Genetic analysis

DNA from whole blood or saliva samples was extracted according to standard procedures as previously described^14^. For linkage analysis for Family (F) 1, DNA from 5 affected and 4 unaffected individuals was genotyped using the IlluminaQC array (15,949 SNPs), individual SNPs were pruned to avoid linkage disequilibrium in PLINK^27^, and multipoint linkage analysis was conducted in MERLIN^28^ with autosomal recessive model with complete penetrance. Haplotypes around candidate regions were constructed manually for F1 and F8 using IlluminaQC array genotyping data. Linkage exclusion analysis was also done for F14 as above, but using an autosomal dominant model with complete penetrance. For exome sequencing, patient samples from three families were combined into equal concentration pools of affected and unaffected family members based on dsDNA fluorescence quantification (QuantiFluor dsDNA System, Promega). Affected patient samples from the F1 families were also pooled for exome sequencing but without an unaffected pool. Sample and library preparation were done at the NIH Intramural Sequencing Center using the xGen Exome capture kit v1 (IDTDNA) and the Illumina NovaSeq Platform. For panel-based sequencing, the NEBNext Ultra II FS DNA Library Prep kit (NEB) and a xGen Lockdown probes (IDTDNA) were used to capture exons and other genomic regions with known or suspected pathogenic variants from a custom 731 genes implicated in eye development or disease (including *MYRF*, *TMEM98*, *PRSS56*, *MFRP, CRB1, BEST1*). These were then sequenced on Illumina MiSeq or NextSeq 550, aligned, variants called, annotated, and prioritized through a custom pipeline available on GitHub (https://github.com/Bin-Guan/NGS_genotype_calling & https://github.com/Bin-Guan/variant_prioritization). The WhatsHap application was used for phasing variants^29^, which were confirmed by direct visualization of aligned sequence reads in the Integrative Genomics Viewer^30^. Copy number variations were called from the panel sequencing data by CoNVaDING^31^. The minor allele fraction differences and presence/absence of a variant between the affected and unaffected pool were used for variant prioritization in the paired-pooled exome sequencing approach.

High quality DNA samples from 52 probands were sequenced by the panel approach. These 52 samples were sequenced with at least 75 × of mean coverage depth of target regions and were included for analysis, with 46/52 sequenced at greater than 200 × mean depth over target regions. Four families were sequenced by pooled exome analysis, with three probands from these families also included on the panel, leading to a total of 53 families for the analysis. All of the exons of known nanophthalmos genes had greater than 10× coverage. Variants were classified based on standardized American College of Medical Genetics (ACMG) criteria^32^. Co-segregation criterion PP1 was applied according to a previous report^33^. Variants were evaluated for the PP3 in-silico prediction criterion using predictions from Varsome^34^, Franklin (https://franklin.genoox.com/), and in-house priority scores as part of our in-house NGS data processing pipeline. The allele frequency of variants in healthy population was acquired from the gnomAD database (https://gnomad.broadinstitute.org/). Designations for solved cases included strict ACMG criteria of two likely pathogenic variants *in trans* for recessive genes, or one likely pathogenic variant. Plausibly solved designation for recessive genes (i.e. *MFRP* or *PRSS56*) was given when there were at least two compelling variants of unknown significance (VUS) or one VUS and a likely pathogenic or pathogenic variant *in trans.* For dominant genes, a single compelling VUS was sufficient for this designation. RefSeq transcript accession numbers used are: *MFRP*, NM_031433.4; *PRSS56*, NM_001195129.1; *TMEM98*, NM_015544.3, *CRB1*, NM_201253.3; *BEST1*, NM_004183.3; *MYRF*, NM_001127392.2. For missense variants within functional domains, homology modeling was performed using the SWISS-MODEL server^35^, and structures were viewed using PyMOL (Schrödinger, LLC, New York, USA). Modeling was based on the following structures: for MFRP, Mannan-binding lectin serine peptidase 2 (5cis); for PRSS56, Mannan-binding lectin serine protease (4kkd); for TMEM98, Cyclin-D1-binding protein 1 (3ay5). Sanger sequencing was used to confirm the DNA sequence variants shown in Table 1 and was used for segregation analysis. Primer sequences are available upon request. The Illustrator for Biological Sequences was used to draw gene diagrams^36^.

**Table 1:** Summary of the genetic findings in this study.

Family ID	Proband accession	Sex	Method	Familial/segregates	Consang?	Ethnicity	Gene	Zygosity	Variants	ACMG class	Solved class
F1	P01131	F	Exome	AR/Y	N	MIXD	*MFRP*	Cmp HET	exon9:c.1022T>C:p.Leu341Pro	Path	S
									exon5:c.498delC:p.Asn167ThrfsTer25	Path	
F2	P02210	M	Panel	S	N	EUWA	*MFRP*	Cmp HET	exon6:c.642-2A>G	LP	S
									exon9:c.1022T>C:p.Leu341Pro	Path	
F3	P05188	M	Panel	S	N	EUWA	*MFRP*	Cmp HET	exon9:c.1090_1091delAC:p.Thr364GlnfsTer27	LP	S
									exon6:c.642-2A>G	LP	
F4	P05208	F	Panel	AR	N	EUWA	*MFRP*	Cmp HET	exon9:c.1124 + 1G>T	LP	PS
									exon7:c.853 T>C:p.Cys285Arg	VUS	
F5	D1108-01	F	Panel	S	N	EUWA	*MFRP*	Cmp HET	exon4:c.313delC:p.Leu105CysfsTer32 exon5:c.629G>T:p.Gly210Val	LP VUS	PS
F6	P04556	M	Panel	S	N	EUWA	*MFRP*	HET	exon9:c.1022 T>C:p.Leu341Pro	Path	U
F7	P05050	F	Panel	S	N	EUWA	*MFRP*	HET	exon8:c.907G>A:p.Gly303Arg	VUS	U
F8	P02228	M	Exome	AR/Y	N	EUWA	*PRSS56*	HOM	exon5:c.506C>A:p.Ala169Glu	VUS	PS
									exon4:c.425C>A:p.Thr142Lys	VUS	
F9	MISC005-1	M	Panel	AR/Y	Y	EUWA	*PRSS56*	HOM	exon9:c.1066delC:p.Gln356ArgfsTer148	LP	S
F10	P04927	M	Panel	F	N	EUWA	*PRSS56*	Cmp HET	exon7:c.849 + 1G>T	Path	S
									exon9:c.1066dupC:p.Gln356ProfsTer152	LP	
F11	P02206	M	Panel	AR	N	EUWA	*PRSS56*	Cmp HET	exon7:c.818G>C:p.Gly273Ala	VUS	PS
									exon8:c.961G>C:p.Val321Leu	VUS	
F12	P05214	F	Panel	S	N	EUWA	*PRSS56*	HET	exon13:c.1651C>T:p.Leu551Phe	VUS	U
F13	P02367	M	Panel	S	N	EUWA	*PRSS56*	HET	exon6:c.661G>A:p.Ala221Thr	VUS	U
F14	P01811	F	Exome	AD/Y	N	EUWA	*TMEM98*	HET	exon7:c.602G>C:p.Arg201Pro	VUS	PS

*ND* not determined, *AR* autosomal recessive, *AD* autosomal dominant, *S* sporadic, *F* familial, *Cmp* compound, *HET* heterozygote, *HOM* homozygote, *VUS* variant of uncertain significance, transcripts used were as follows: *MFRP* , NM_031433.4; *PRSS56*, NM_01195129.1; *TMEM98* , NM_001033504; *ACMG* American College of Medical Genetics, *Path* pathogenic, *LP* likely pathogenic, *VUS* variant of unknown significance, *S* solved, *PS* plausibly solved, *U* unsolved.

### Web resources

gnomAD Database: https://gnomad.broadinstitute.org/. NCBI Human Reference Genome Build 37.1: https://www.ncbi.nlm.nih.gov/genome/assembly/2928/. GitHub (https://github.com/Bin-Guan/NGS_genotype_calling & https://github.com/Bin-Guan/variant_prioritization). Varsome: https://varsome.com/. Franklin (https://franklin.genoox.com/).

## Results

### Novel and recurrent MFRP, PRSS56, and TMEM98 variants in nanophthalmos

To define the prevalence of variants in known genes *PRSS56, MFRP, TMEM98, MYRF*, we systematically collected DNA samples from 53 families, excluding any with other known genetic diagnosis or poor quality DNA. Of this patient cohort, 46 families met the nanophthalmos criteria of ≤ 21.0 mm axial length, while 7 met the criteria of solely high hyperopia with SE refractive error ≥  + 5.50 (Figure S1). Among these, 39 were sporadic cases, while 14 were familial, with either probable autosomal recessive (9) and dominant (5) inheritance patterns and only one family (F9) with known consanguinity. DNA from patient samples from three large families was used for SNP analysis for linkage exclusion and haplotype analysis, while the remaining families underwent panel-based next-generation sequencing. Plausibly disease-causing variants in *MFRP*, *PRSS56*, and *TMEM98* were identified in 10/53 families (18.9%, Table 1), but with strict ACMG criteria only 5/53 (9.4%) were considered definitively solved. Leber congenital amaurosis gene *CRB1* and macular dystrophy gene *BEST1* have very distinctive clinical phenotypes^21,22^ in addition to small eyes, but were also included in our panel in order to determine if genetic variants might lead to atypical phenotypes. We identified one variant of unknown significance in the *CRB1* gene, NM_201253.3:c.443A>T p.(Asp148Val), but this was not thought to be causative, as this residue is poorly conserved and the patient phenotype does not fit with that seen in *CRB1*-associated disease. No plausible disease-causing variants were observed in *MYRF* exonic sequence, as expected based on prior Sanger screening^14^.

**Figure 1:**
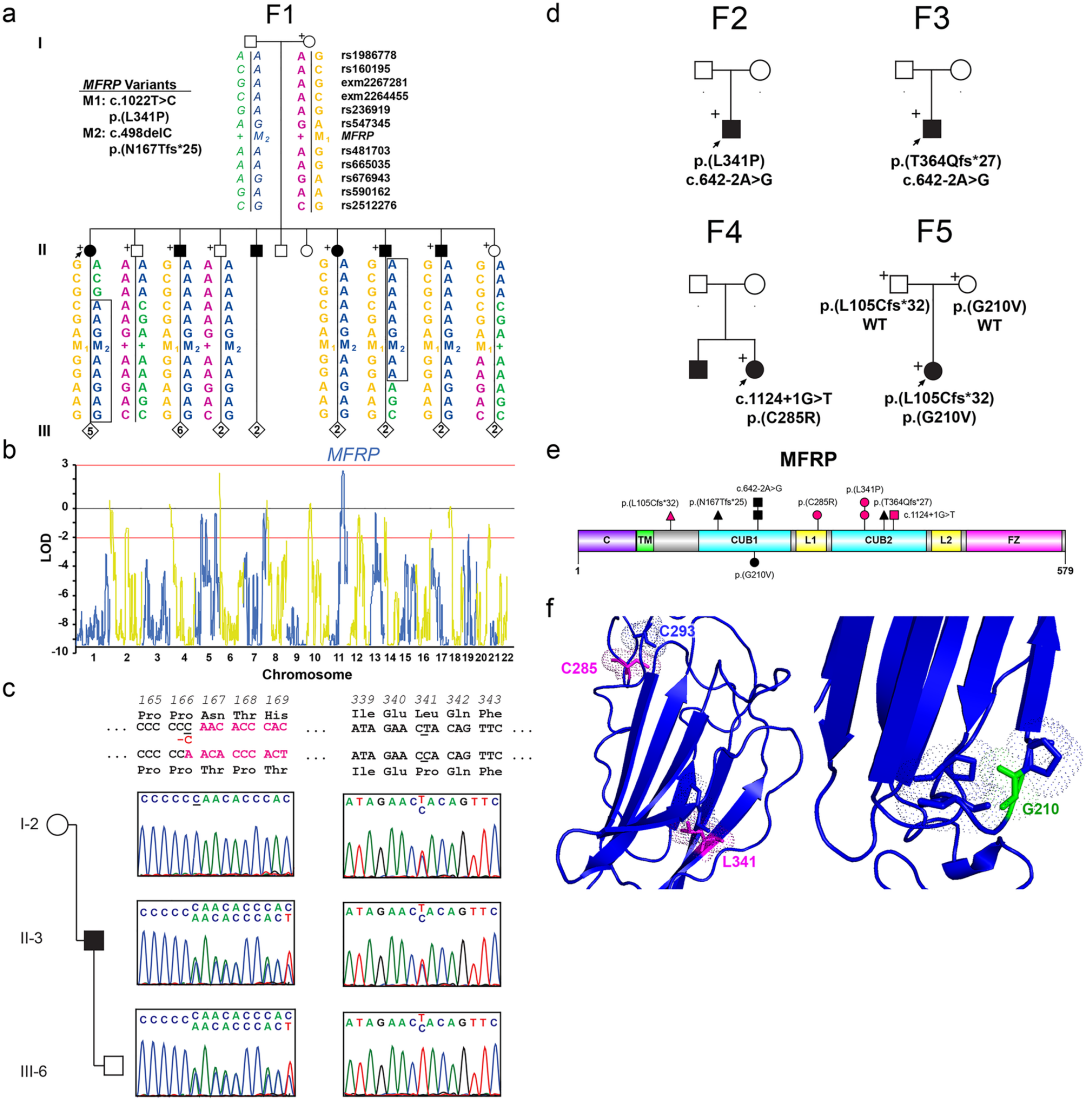
Genetic features of solved *MFRP* families. (**a**) Pedigree and haplotype analysis of F1 nanophthalmos family carrying *MFRP* variants (M1,M2). Boxes mark recombinant individuals. The minimal non-recombinant interval is bounded by exm2267281 and rs676943 and includes the *MFRP* gene. (**b**) Whole genome multi-point linkage analysis of F1 showing peaks on chr 6p and 11q with suggestive linkage. The chr11q peak contains the *MFRP* gene. (**c**) Sequencing chromatograms showing presence of both *MFRP* variants (c.498delC and c.1022T>C) in affected individuals, and only one variant in unaffected parents or children. (**d**) Pedigrees and variants identified in *MFRP* based on transcript NM_031433.4. Samples available for genotyping are included with their genotypes. (**e**) Protein diagram of *MFRP* showing location of all identified variants in this study. Frameshifts (triangle), splice altering (square), and missense (circle) variants are marked, with magenta being variants newly described in this study. (**f**) Homology modeling of missense variants in *MFRP* showing critical interactions of C285, L341, and G210. +, sampled individuals; arrow, proband.

Genetic analysis revealed novel and recurrent variants in *MFRP* in 5/53 families (9.4%, Fig. 1, Table 1). Among *MFRP* families, F1 showed suggestive linkage of the nanophthalmos trait to chromosome 11q with max parametric LOD score of 2.61, theta = 0 (Fig. 1b). Haplotype analysis revealed a minimal nonrecombinant interval bounded by exm2267281 and rs676943 (34 MB interval), which encompassed the *MFRP* gene. Pooled exome sequencing from all of the affected family members revealed two pathogenic *MFRP* variants (c.498delC p.(Asn167Thr*fs*Ter25) and c.1022T>C p.(Leu341Pro)). Segregation analysis showed compound heterozygosity for these variants in each diseased individual, while sampled individuals with one pathogenic variant had normal axial length and refraction (Fig. 1c). The frameshift c.498delC was a known disease associated variant, and previously identified in 7 families^15,25,37–40^, while the c.1022T>C p.(Leu341Pro) was a previously undescribed variant that is present at a very low frequency in gnomAD (6/248,908, 2.4 × 10^–5^), predicted to be damaging by SIFT, Polyphen, and Clinpred in silico tools, and was also identified in two other sporadic cases: F2 (P02210) and F5 (P04456) (Fig. 1d, Table 1). Homology modeling revealed that this variant is likely to disrupt a conserved beta-sheet in the CUB domain and expected to destabilize the protein (Fig. 1e,f). Additional biallelic *MFRP* variants identified in this study included a novel recurrent canonical splice disrupting variant c.642-2A>G, a previously reported c.1124 + 1G>T splice disrupting variant^24,41,42^, another previously described frameshift variant c.1090_1091del p.(Thr364Gln*fs*Ter25)^25^, and two missense variants c.853T>C p.(Cys285Arg) and c.629G>T p.(Gly210Val). One family had a single pathogenic missense variant in *MFRP* p.(Leu341Pro), and an additional family had a single variant of unknown significance (VUS): *MFRP* p.(Gly303Arg). We did not detect any split reads and read depth was comparable to remaining portions of the gene, suggesting no exon level copy number variants in these two individuals. Of the *MFRP* missense variants, homology modeling of Cys285 revealed that this residue likely forms a disulfide bond with Cys293, and disruption of this residue would grossly affect protein folding (Fig. 1f). This *MFRP* p.(Cys285Arg) missense variant of uncertain significance was found to be *in trans* with the pathogenic canonical splice donor disrupting variant c.1124 + 1G>T variant (Fig. 1d). The p.(Gly210Val) *MFRP* variant was present in *trans* with a disruptive frameshift variant p.(Leu105Cys*fs*Ter32), and has previously been characterized as a VUS in ClinVar probably due to its high allele frequency in gnomAD 0.4%. This residue has Van der Waals interactions with nearby residues, and introduction of a larger amino acid side chain (valine) would be expected to alter folding of the hinge region of the CUB domain.

**Figure 2:**
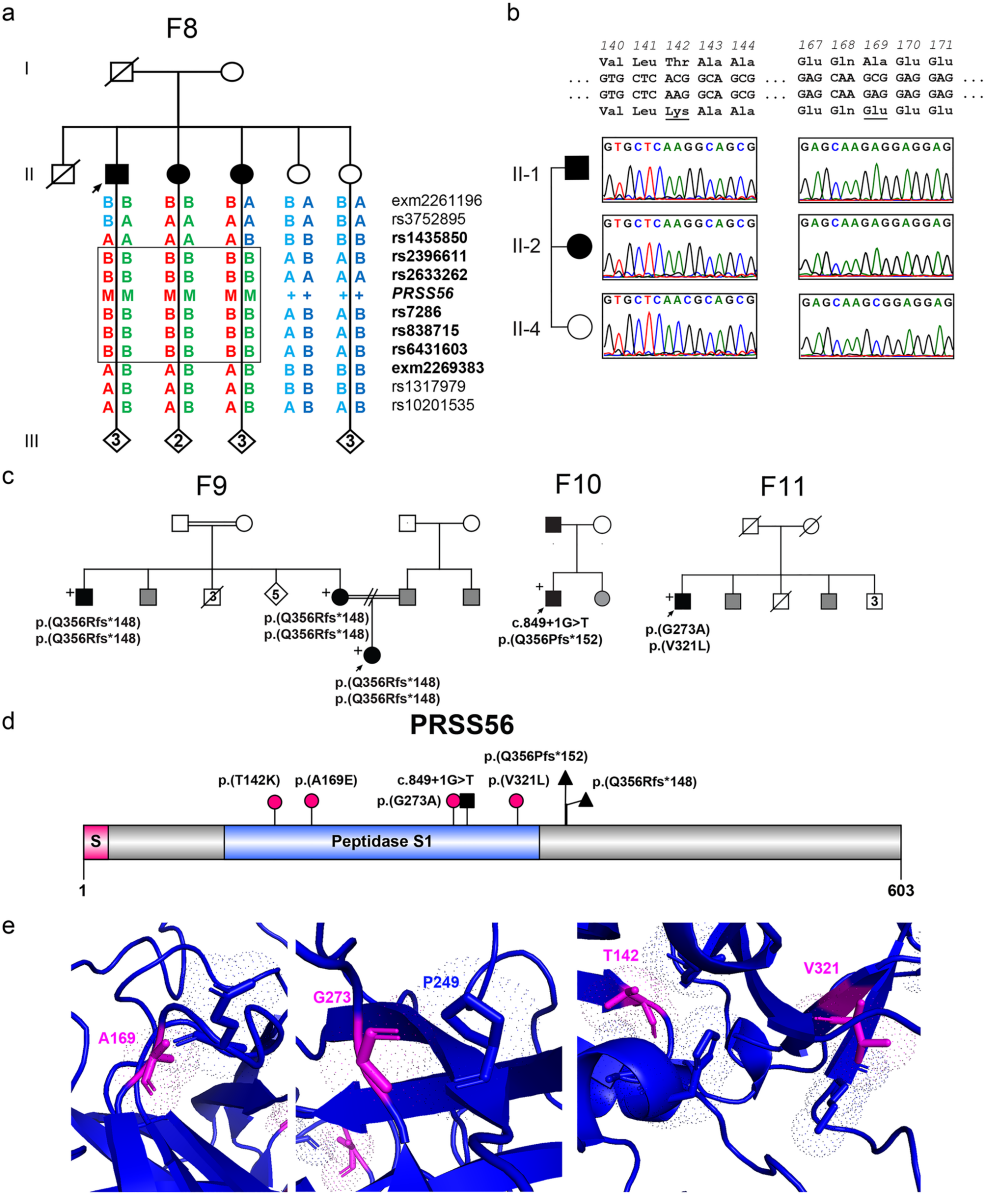
Genetic features of solved *PRSS56* families. (**a**) Pedigree and haplotype analysis of F8 showing minimal area of homozygosity bounded by rs1435850 and exm2269383. Major allele is marked as A and minor allele marked as B, with *PRSS56* variant indicated by M, and proband indicated by the arrow. (**b**) Sequencing chromatograms showing segregation of two missense p.(T142K) and p.(A169E) variants among all affected individuals. (**c**) Pedigrees and variants identified in other solved families. Black shading indicates phenotyped affected individuals, while gray shading indicates familial report of affected status or thick glasses. (**d**) Protein diagram of *PRSS56* showing location of all identified variants in this study. Frameshifts (triangle), splice altering (square), and missense (circle) variants are marked; magenta shows newly described variants. (**e**) Homology modeling of *PRSS56.* + , sampled individuals.

Genetic analysis uncovered 4/53 families with two likely deleterious *PRSS56* variants, and an additional three families with single variants in this gene. SNP analysis of F8 showed an 11 MB minimal region of homozygosity bounded by rs1435850 and exm2269383 surrounding *PRSS56* (Fig. 2a), and exome sequencing revealed homozygosity for two missense variants in the affected pool: c.506C>A p.(Ala169Glu) and c.425C>A p.(Thr142Lys). We did not detect any split reads and read depth in this region was comparable to coverage in remaining portions of the gene and for other sequenced samples. Together, these suggest that there was no microdeletion and that both variants were homozygous in all affected individuals. The variants were absent in unaffected siblings (Fig. 2b). The p.(Ala169Glu) variant is absent in gnomAD, while p.(Thr142Lys) is present at a very low level (6/143,888,4 × 10^–5^). Homology modeling revealed that both variants have Van der Waals interactions with nearby residues (Fig. 2e). Substitution for charged amino acids, lysine or glutamic acids, would be expected to alter these interactions. As both of these are classified as VUS under ACMG criteria, it is unclear which is contributing to disease or if both together play a functional role. Additional novel rare *PRSS56* missense variants were identified in F11: c.818G>C p.(Gly273Ala), and c.961G>C p.(Val321Leu), which are both absent in gnomAD and predicted to be deleterious based on in silico analysis (Table S1) and on homology modeling (Fig. 2e). Additional identified variants included a described canonical splice donor variant (c.849 + 1G>T)^24^, and two previously described frameshift variants: c.1066delC p.(Gln356Arg*fs*Ter148) and c.1066dupC p.(Gln356Pro*fs*Ter152)^24,25^ (Fig. 2c,d). Two additional probands carried single *PRSS56* variant alleles i.e. p.(Leu551Phe) and p.(Ala221Thr) (Table 1), and no exon level deletions or duplications were identified based upon split-read or read depth analysis.

**Figure 3:**
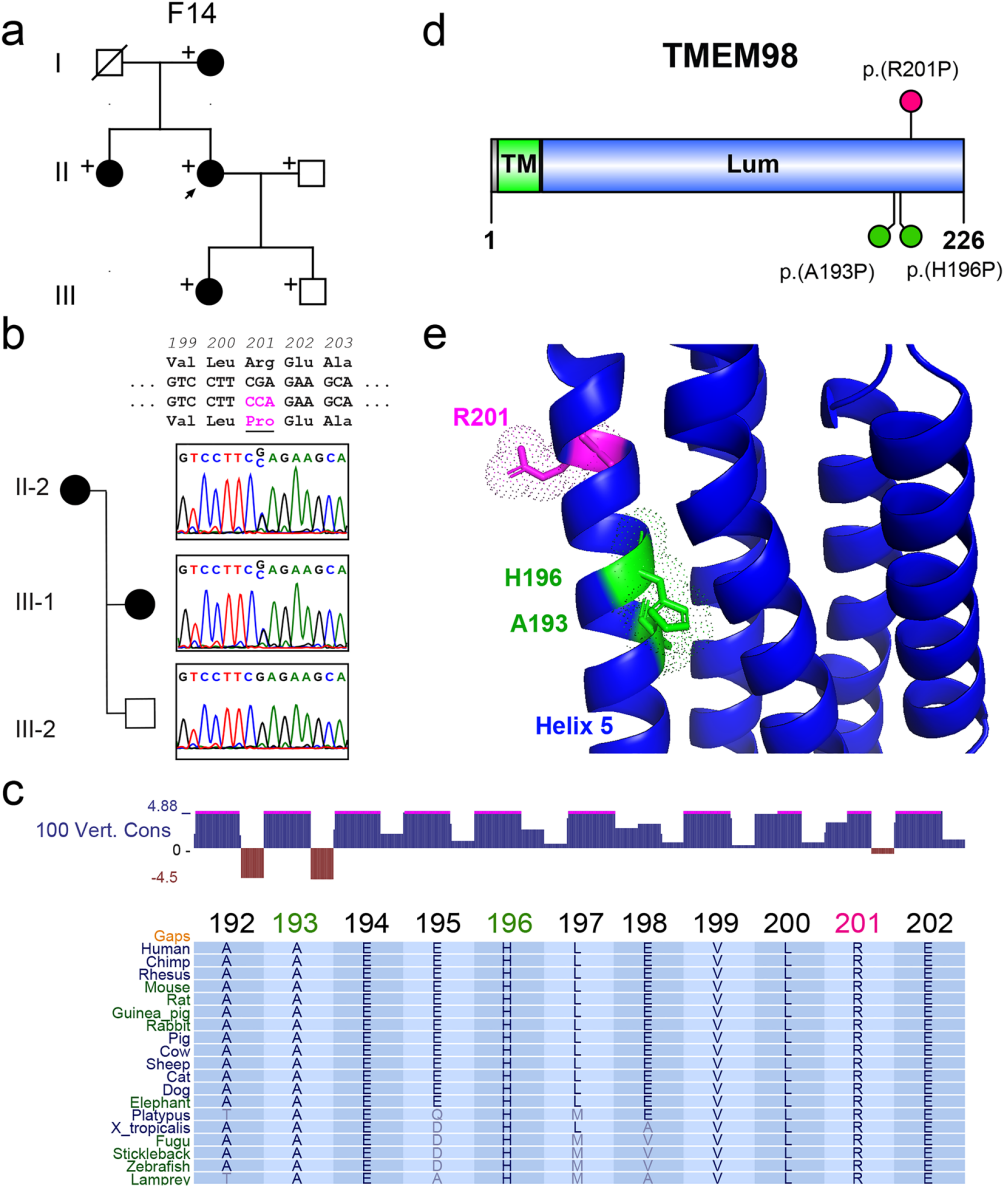
Novel *TMEM98* variant segregates with nanophthalmos in a 3-generation pedigree. (**a**) Three generation pedigree showing 4 affected individuals with nanophthalmos. (**b**) Sequencing chromatograms showing identified c.602G>C:p.(R201P) variant present in affected individuals, but not unaffected family members. (**c**) UCSC browser plot of 100 vertebrate conservation and MultiZ alignment of TMEM98 showing complete conservation of R201, as well as prior disease associated residues A193 and H196 down to lower vertebrate animals such as zebrafish. (**d**) Protein diagram of TMEM98 showing location of our identified variant p.(R201P) (magenta), and previously identified disease associated variants (green). (**e**) Homology modeling of TMEM98 based on Cyclin-D1-binding protein 1 crystal structure (3ay5.1). + , sampled individuals, arrow, proband.

Using exome sequencing, we identified a novel heterozygous missense *TMEM98* variant in F14: c.602G>C p.(Arg201Pro) (Fig. 3). This family shows autosomal dominant inheritance (Fig. 3a) and the variant segregates perfectly with the disease phenotype (Fig. 3b). This variant is highly conserved, predicted to be damaging in silico, and absent in gnomAD (Fig. 3c, Table 1). This variant introduces a proline into helix 5 of the luminal domain of *TMEM98*, similar to two other identified disease variants p.(Ala193Pro) and p.(His196Pro)^18,19^ (Fig. 3d), which would be expected to disrupt the α-helix (Fig. 3e). We classified this variant as a VUS considering PM2 (absent in gnomAD), moderate evidence of co-segregation (PP1 > M), and PP3 (in silico). However, clinical correlation, in silico and co-segregation evidence is strongly suggestive of *TMEM98* variant as the plausible cause of nanophthalmos in this family.

### Clinical phenotyping of nanophthalmos/high hyperopia families

**Figure 4:**
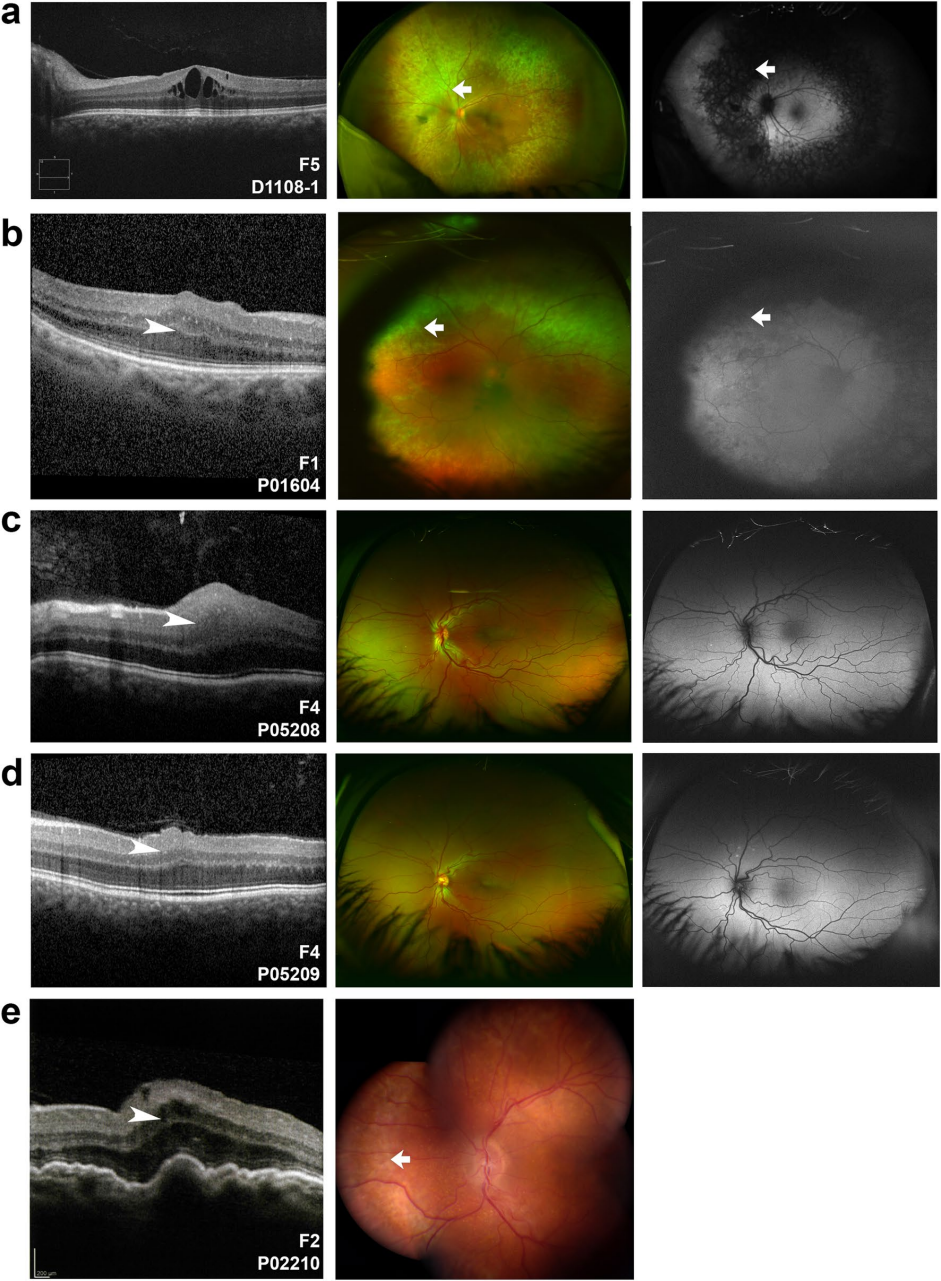
Clinical imaging features of *MFRP* families. (**a**–**e**) OCT (left), Optos wide field photographs (**a**–**d**, middle) or composite fundus photograph (**e**, middle), and Optos fundus autofluorescence imaging of patients with biallelic *MFRP* variants (**a**–**d**, right). Three patients (**a**,**b**,**e**) had evidence of retinal degeneration in a characteristic ring pattern of atrophy (arrows). Two siblings (**c**,**d**) had very similar clinical appearance with macular folds (arrowheads), foveal hypoplasia, punctate hyperautofluorescent white lesions, crowded discs and vascular tortuosity. P02210 had prominent choroidal folds and foveoschisis (**e**).

**Figure 5:**
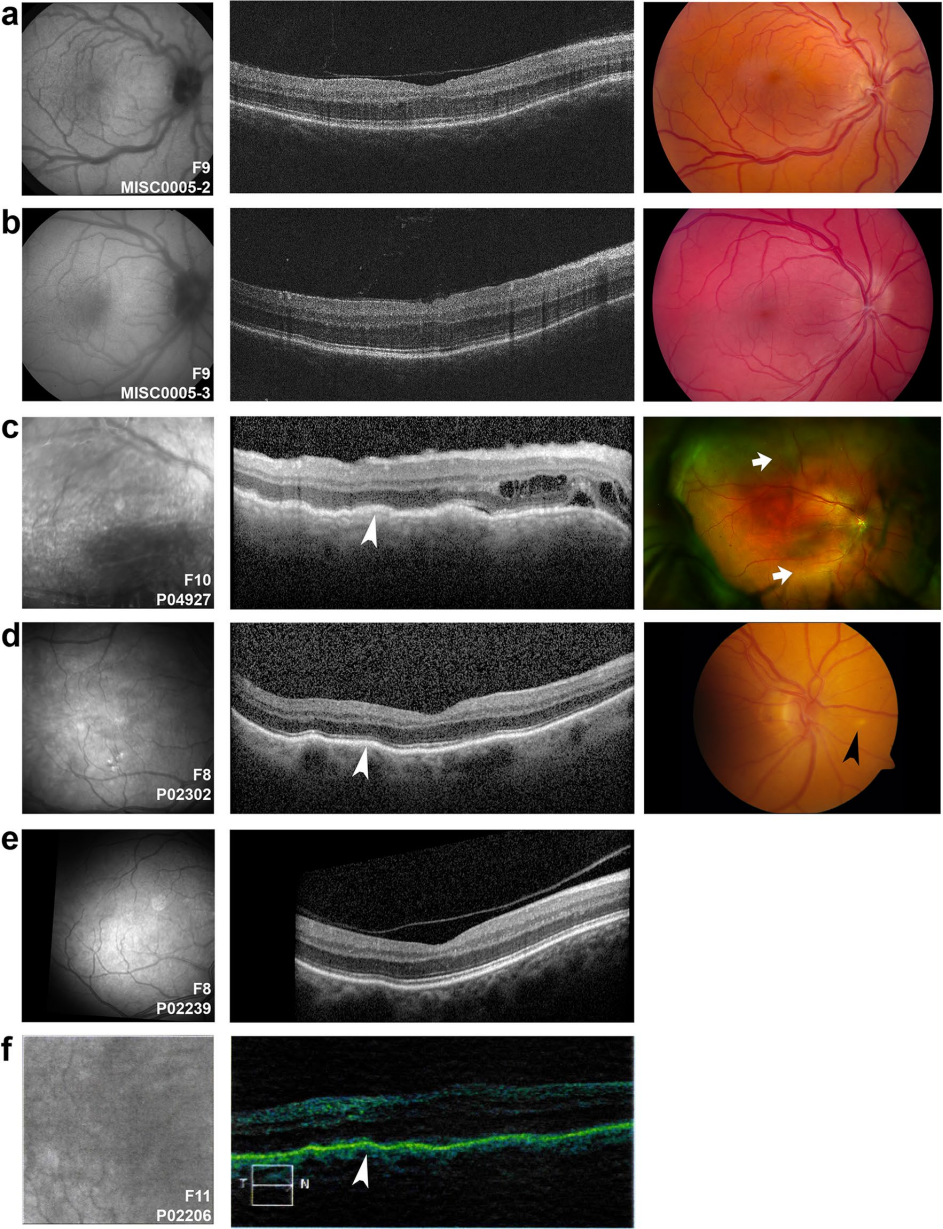
Clinical imaging features of *PRSS56* families. (**a**,**b**) TopCon fundus autofluorescence (left) and color (right) and OCT (middle) images showing mild hypoautofluorescence in the macula, crowded discs, vascular tortuosity, and foveal hypoplasia among two family members carrying PRSS56 deleterious variants (MISC0005-2, F10, **A**) and (MISC0005-3, F10, **B**). (**c**–**f**) Infrared reflectance (left) and corresponding OCT image (middle) of patients carrying *PRSS56* biallelic variants, along with Optos wide-field imaging (**c**, right) or optic disc image (**d**, right). P04927 (F11) has a serous retinal detachment along with intraretinal fluid (**a**, arrow) and pigmentary changes. Siblings P02302 and P02239 from F9 have similar clinical appearance with choroidal folds (white arrowheads) and mild foveal hypoplasia, with P02302 having white lesions in the retina (black arrowhead). These patients have prominent choroidal folds (**c**–**f**).

**Figure 6:**
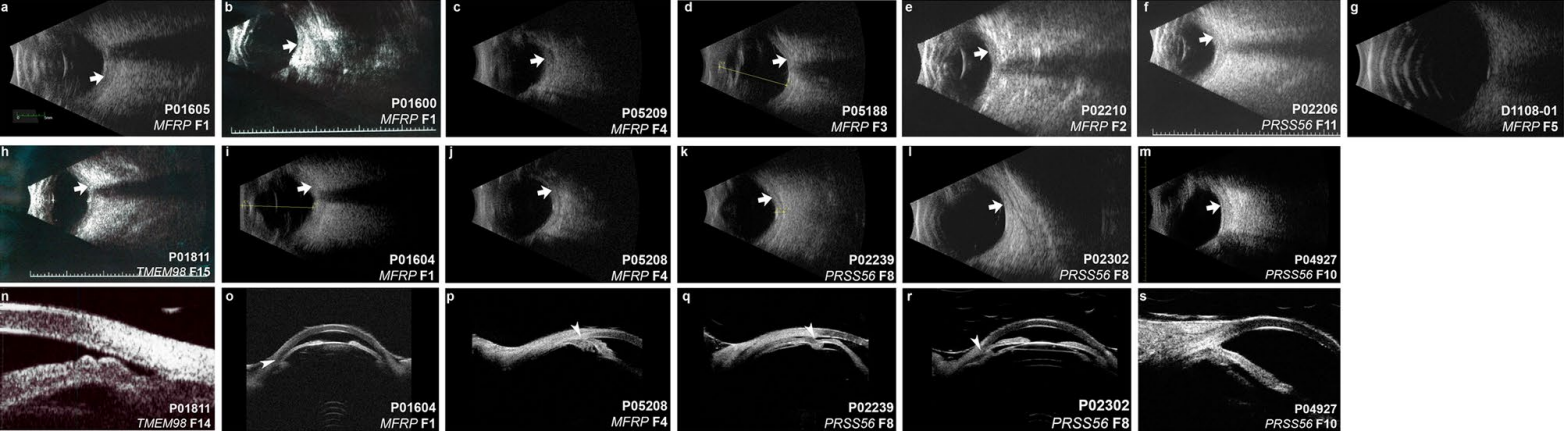
Ultrasonographic features of solved nanophthalmos/high hyperopia families. (**a**–**m**) B-scan ultrasound of solved families. There is variability in eye size within and among families, with consistent features of increased sclerochoroidal thickness (arrows). (**n**–**r**) Ultrasound biomicroscopy (UBM) of solved families. Plateau iris (**n**), and angle closure (**o**–**r**, arrowheads) are consistent features among families. There are no clear distinctions on B-scan or UBM among different genetic causes.

Twenty-seven individuals from the 10 solved families had available clinical phenotypic data (Figs. 4, 5, 6 and Table S2). Distinctions between posterior microphthalmos and nanophthalmos were not routinely made given that these conditions are often allelic and have presumedly overlapping pathogenesis. However, anterior chamber depth and angle pathology were documented by ultrasound biomicroscopy and/or gonioscopy when available. Overall, a higher fraction nanophthalmos cases were plausibly solved (10/46, 22%) as compared to cases with solely high hyperopia (0/7) (Figure S1). Likewise, 6/14 (43%) of familial cases were solved, whereas only 4/39 (10%) sporadic cases were solved (Figure S1). Best corrected visual acuity ranged from logMAR 0 to no light perception (NLP) with median logMAR 0.5, and several patients had experienced complications secondary to cataract surgery as previously described^26^.

Among *MFRP* families, four patients were noted to have retinal degeneration (Fig. 4) with a characteristic pattern of retinal atrophy on OCT, white dots on fundus photograph and a ring pattern of hypoautofluorescence on fundus autofluorescence (Fig. 4a,b,e), similar to those observed in other studies^25^. Two siblings (G5208 and P05209 from F4) had no evidence of retinal degeneration, but several small foci of hyperautofluorescence with strikingly similar clinical appearance among both siblings (Fig. 4c,d). All examined patients had variable foveal hypoplasia, with three having some evidence of macular edema or foveoschisis (D1108-1, P01604, P02210, Fig. 4a,b,e) and only one having choroidal folds (Fig. 4e). The range of axial length for affected individuals was 13.6–19.8 mm (median 16.1 mm, Table S2), while refractive error ranged from + 8.4 D SE to + 24.3 D SE (median + 15.8 D). Anterior segment exam was notable for narrow iridocorneal angles in all examined phakic patients.

Among *PRSS56* families, choroidal folds and foveal hypoplasia were a common finding in all imaged individuals (Fig. 5). Additionally, serous retinal detachment (Fig. 5c) or white retinal lesions (Fig. 5d) were present in some imaged individuals. Three individuals had evidence of pigmentary retinopathy, but this was in the context of prior uveal effusion syndrome or retinal detachment (Fig. 5c, Table S2). All examined individuals had narrow angles, and two individuals had aqueous misdirection. The range of axial length for affected individuals was 15.1–17.5 mm (median 16.3 mm, Table S2), while refractive error ranged from + 9.4 D SE to + 25.0 D SE (median 13.9 D). Fundus imaging was not available for *TMEM98* family members, but refractions were in the range + 8.9 to  + 10.3 D SE (median =  + 9.9 D), though individuals still had evidence of narrow angle and glaucoma (Table S2). Median phakic refractions for unsolved families were + 6.8 D SE (range: + 2.25 to  + 11.5 D) and median axial length was 20.5 mm (range 16.75–22.03 mm), when including non-qualifying eyes of qualifying patients.

Ultrasonography revealed increased peripapillary choroidoscleral thickness in all imaged individuals (Fig. 6a–m), though precise measurements were done in only some of the patients due to imaging quality. There was no qualitative difference in choroidoscleral thickness among individuals based on genotype. UBM revealed shallow anterior chambers and narrow angles in all examined phakic patients (Fig. 6). Other qualitative features included anterior rotation of the ciliary body consistent with plateau iris configuration in some individuals (Fig. 6n,q,s). Two subjects were pseudophakic at the time of UBM (Fig. 6q,r), with one having a deep anterior chamber, open angle, but anterior rotation of the ciliary body (Fig. 6q). Anterior chambers were shallow in all affected individuals, who were phakic with their natural crystalline lens, and there were no distinctive phenotypic differences based on genotype.

## Discussion

We have defined the diagnostic yield of sequencing the coding variants in *PRSS56, MFRP*, *TMEM98, MYRF, CRB1, and BEST1* in a large cohort of patients with nanophthalmos and high hyperopia. These include 4 newly identified *PRSS56* variants, 4 newly identified *MFRP* variants, and 1 new *TMEM98* variant. Together, this a comprehensive evaluation of sporadic and familial cases of high hyperopia and nanophthalmos for rare genetic variants.

We defined a novel *TMEM98* variant p.(Arg203Pro), which segregates with nanophthalmos in one family. Interestingly, all three identified missense variants in *TMEM98* have involved proline substitution within helix 5 of the protein, which would be expected to alter the folding of this region of the protein. TMEM98 interacts with MYRF (another nanophthalmos gene product) in the retinal pigment epithelium (RPE) and oligodendrocytes in the brain^8,14,43^, though the interaction domain is dependent on the N-terminus of TMEM98 and not these C-terminal binding regions^44^. However, it is possible that a protein–protein interaction between TMEM98 and another protein leads to eye specific disease. It is tempting to speculate that this interaction would rely on helix 5 of the TMEM98 luminal domain.

There is a significant disparity in the relative contribution of genes to nanophthalmos pathogenesis among prior reports. In a study of mostly consanguineous pedigrees, biallelic *MFRP* and *PRSS56* were found in 18/21 families (85%)^25^, while in Chinese high hyperopia cohorts *MFRP* and *PRSS56* were identified 6 and 8%, respectively^45,46^. *TMEM98* is a very rare cause of nanophthalmos, with only three reported families^18,19^, while *MYRF* can be associated with rare syndromic or predominantly ocular forms of the condition^14,47–49^. Our data for the United States population suggest an intermediate estimate. Our study has several distinctions from other described cohorts which may explain the difference in prevalence. First, our cohort only includes one family with known consanguinity, whereas others have been described in founder populations or consanguineous unions^16,25^. In conditions such as primary congenital glaucoma, consanguinity strongly biases the solved rate, with *CYP1B1* variants explaining only 15% of cases in a United States cohort^50^ versus 92% of Saudi Arabian consangeous familial cases^51^. Second, our cohort was collected primarily based on ascertainment of refraction or axial length within a large number of patients seen at the National Eye Institute and the University of Michigan. We do not include genetic analysis of previously solved families, which could introduce additional ascertainment bias. Third, we include a wider range of axial lengths and refractive errors than previous reports. When considering stringent axial length cutoffs, our rate of plausibly solved cases increases to 69% (9/13) in patients with AL < 18 mm, and 40% (10/25) when AL < 20 mm (Figure S1). Likewise, our plausibly solved rate for familial nanophthalmos cases (60%) is higher than the rate for cases overall, suggesting familial cases are more likely to be explained by variants in known genes (Figure S1). These results suggest that forms of nanophthalmos with the most extreme axial lengths are most likely to be explained by variation in *PRSS56* and *MFRP*. Fourth, when we use strict ACMG criteria, our solved rate (including two likely pathogenic or pathogenic variants in recessive genes or one pathogenic or likely pathogenic variant in a dominant gene) decreases substantially. With these criteria 3/10 (30%) of familial cases, 5/53 (9.4%) are solved. These strict criteria are necessary for accurate results return to patients, and future genetic and functional studies may allow us to reclassify many of the plausibly solved cases into solved ones.

In addition to uncovering likely disease-causing variants, we identified 5 families with single deleterious variants in recessive genes *PRSS56* and *MFRP*. In families with biallelic *MFRP* pathogenic variants, heterozygous carriers have a normal axial length and refraction both in our study (F1 for example) and previously^15^, suggesting that these patients with single deleterious alleles in *MFRP* either have an additional regulatory or deep intronic mutation or another genetic cause for their nanophthalmos. Importantly, our NGS panel analysis excludes the possibility of exon level deletions within the gene or other copy number variation in these cases, through uniform coverage coupled with read depth and split-read analysis. For *PRSS56*, the situation is less clear, as the refraction status and biometry of heterozygous carriers has not been explored in detail. Interestingly, *PRSS56* has been implicated in GWAS for hyperopia and myopia suggesting a role in eye size^23^. As such, gene dosage of *PRSS56* may have a semi-dominant effect. Single hit *PRSS56* probands had higher axial lengths than those with 2 deleterious variants, suggesting the possibility that gene dosage of *PRSS56* may have a more general role in controlling refractive error and axial length. Alternatively, undiscovered regulatory variants in *PRSS56* may alter expression of the wild-type copy leading a threshold effect in these patients.

While there was significant phenotypic variability between families with each genetic diagnosis and no strict genotype–phenotype correlations, several trends were evident. First, family members carrying the same variants showed strikingly similar retinal phenotypes (Figs. 4c,d and 5b,c) regardless of the gene. Second, *MFRP* families were split into those with a stereotypical retinal degeneration (Fig. 4a,b,e) and those without retinal degeneration (Fig. 4c,d), but this did not correlate with the type or nature of the variants. *PRSS56* families had overt retinal degeneration only in the context of surgical complication, i.e. serous retinal detachment (Table S2, Fig. 5a), and the retinal degeneration in these cases had a different appearance than in the *MFRP* cases. Third, the axial lengths of solved cases were shorter than in unsolved cases, similar to that seen previously^24^. Fourth, common clinical features regardless of genotype included narrow angles in all phakic patient in which angle anatomy was evaluated and mild foveal hypoplasia (Grade I).

Our study explored the genetic basis of nanophthalmos and high hyperopia in a large United States cohort and identified genetic diagnoses in only 10/53 cases (19%). There remain over 80% of sporadic and familial cases that are not explained by variation in the known nanopthalmos genes, suggesting opportunities for additional gene discovery. Given the strong heritability of hyperopia, these cases are likely explained by either multiple genes, yet undiscovered genes, regulatory variants in known genes, or gene-environment interactions. Uncovering these additional genetic and molecular pathways will lead to significant insights into the growth and development of the eye and may ultimately improve our clinical care for these complex patients.

## Supplementary information

Supplementary Information

## Data Availability

Underlying data presented in the manuscript are available from the corresponding authors at the reasonable request.
