# Supplementary material for: Novel TMEM98, MFRP, PRSS56 variants in a large United States high hyperopia and nanophthalmos cohort
Source: Sci Rep. 2020 Nov 17;10:19986. doi: 10.1038/s41598-020-76725-8 (PMC7672112; doi:10.1038/s41598-020-76725-8)
Supplement: Supplementary file 1 — Supplementary Information [file 41598_2020_76725_MOESM1_ESM.pdf]

Novel *TMEM98, MFRP, PRSS56* variants in a large United States high hyperopia and nanophthalmos cohort

Lev Prasov[1,2*], Bin Guan[3], Ehsan Ullah[3], Steven M. Archer[1], Bernadete M. Ayres[1], Cagri G. Besirli[1], Laurel Wiinikka-Buesser[1], Grant M. Comer[1], Monte A. Del Monte[1], Susan G. Elner[1], Sarah J. Garnai[1], Laryssa A. Huryn[3], Kayla Johnson[1], Shivani S. Kamat[1], Philip Lieu[1], Shahzad I. Mian[1], Christine A. Rygiel[1], Jasmine Y. Serpen[1,3,5], Hemant S. Pawar[1], Brian P. Brooks[3], Sayoko E. Moroi[1,4], Julia E. Richards[1], Robert B. Hufnagel[3,*]

[1] Department of Ophthalmology and Visual Sciences, University of Michigan, Ann Arbor, MI 48105

[2] Department of Human Genetics, University of Michigan, Ann Arbor, MI 48109

[3] Ophthalmic Genetics and Visual Function Branch, National Eye Institute, National Institutes of Health, Bethesda, MD 20892

[4] Department of Ophthalmology and Visual Sciences, The Ohio State University, Columbus, OH 43212

[5] Case Western Reserve University School of Medicine, Cleveland, OH, 44106

* Authors for correspondence:
Lev Prasov, MD, PhD
229 Kellogg Eye Center
1000 Wall St.
Ann Arbor, MI 48105
Tel 734-763-3732
lprasov@med.umich.edu

Robert B. Hufnagel, MD, PhD
10 Center Drive
Bldg 10, rm 10N109
Bethesda, MD 20892
Tel 301-503-1305
Robert.hufnagel@nih.gov

Running Title: Small eye genetics

Key Words:

Nanophthalmos	Pooled exome sequencing	Small eye
TMEM98	Angle closure glaucoma	Retinal degeneration
MFRP	Retinal pigment epithelium (RPE)	High hyperopia
BEST1	*PRSS56*	Posterior microphthalmos
CRB1		

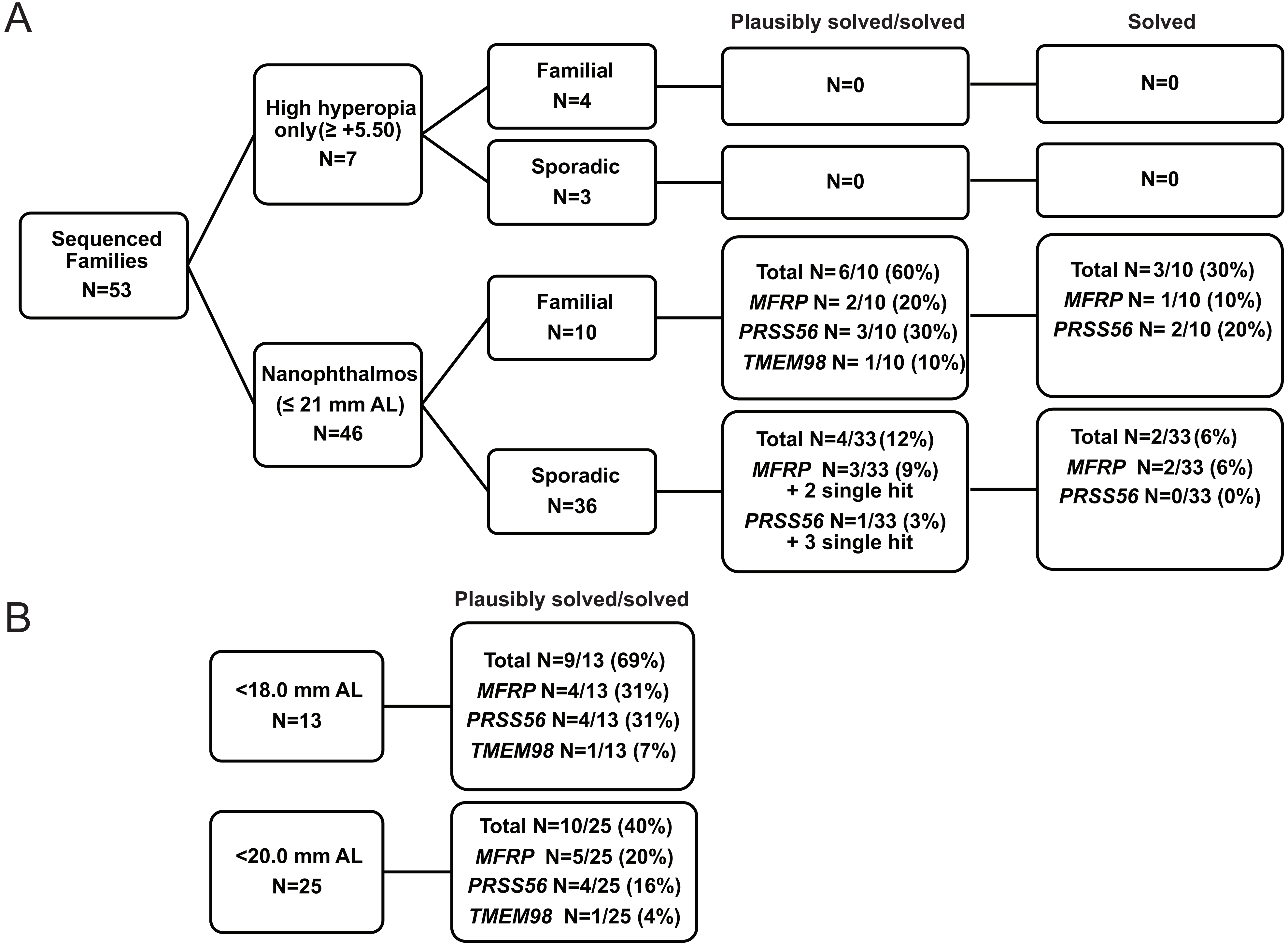

Figure S1: Fractions of solved cases by genotype and phenotype. A) Flowchart showing fractions of solved cases among high hyperopes, nanophthalmos sporadic and familial cases. B) Flowchart based on stringent definitions of nanophthalmos from prior reports. A high fraction of families was solved among those with the most significantly reduced axial lengths (<18 mm).

Table S1: Categorization of variant level data among nanophthalmos and high hyperopia cases

hg19 variant ID	Gene	HGVSc	HGVSp	gnomAD AF	In-house total	In-house *in silico* score	*in silico* (Varsome)	*in silico* (Franklin, Aggregated Prediction)	ClinVar	HGMD	HGMD Publications	Zygosity	Segregation	ACMG Evidence	ACMG Class	Reference
11-119214525-C-A	*MFRP*	NM_031433.4:c.1124+1G>T	NA	0.00006065	17.1	2.5	dbscSNV ADA 0.9999, RF 0.934	splice AI Exome and Genome : Splice Altering (	Uncertain	DM	2	Het	in trans by whatsh	PVS1, PM2	Likely Pathogenic	Siggs et al., 2020; Wang et al., 2014; Dinculescu et al., 2012
11-119214558-GGT-G	*MFRP*	NM_031433.4:c.1090_1091delA(	p.Thr364GlnfsTer27	0.00005221	11.5	0.5	NA	NA	Absent	Absent	NA	Het	phase not determi	PVS1, PM2	Likely Pathogenic	This study
11-119214628-A-G	*MFRP*	NM_031433.4:c.1022T>C	p.Leu341Pro	0.00002411	11	5	Pathogenic computational verdict because 6 pathogenic predictions from DANN, EIGEN, FATHMM-MKL, M-CAP, MutationAssessor and MutationTaster vs 3 benign predictions from DEOGEN2, MVP and REVEL.	Uncertain (0.43)	Absent	Absent	NA	Het	in trans by familial segregation	PS4-moderate, PM2, PM3, PP3, PP1-strong	Pathogenic	This study
11-119215093-C-T	*MFRP*	NM_031433.4:c.907G>A	p.Gly303Arg	Absent	11	5	Pathogenic computational verdict because 6 pathogenic predictions from DANN, EIGEN, FATHMM-MKL, M-CAP, MutationAssessor and MutationTaster vs 3 benign predictions from DEOGEN2, MVP and REVEL.	Uncertain (0.31)	Absent	Absent	NA	Het	NA	PM2, PP3	VUS	This study
11-119215387-A-G	*MFRP*	NM_031433.4:c.853T>C	p.Cys285Arg	0.00003216	12	5	Pathogenic computational verdict because 11 pathogenic predictions from DANN, DEOGEN2, EIGEN, FATHMM-MKL, M-CAP, MVP, MutationAssessor, MutationTaster, PrimateAI, REVEL and SIFT vs no benign predictions.	Deleterious (0.96)	Absent	Absent	NA	Het	in trans by whatsh	PM2, PM3, PP3	VUS	
11-119215716-T-C	*MFRP*	NM_031433.4:c.642-2A>G	NA	0.0001046	21.5	2.5	dbscSNV ADA 0.9999, RF 0.9459	splice AI Exome and Genome : Splice Altering (	Conflicting	Absent	NA	Het	phase not determi	PVS1, PM2	Likely Pathogenic	This study
11-119216142-C-A	*MFRP*	NM_031433.4:c.629G>T	p.Gly210Val	0.003819 (03 hm	8	4	Benign computational verdict because 6 benign predictions from DEOGEN2, EIGEN, MVP, MutationAssessor, PrimateAI and REVEL vs 4 pathogenic predictions from DANN, FATHMM-MKL, MutationTaster and SIFT and the position is not conserved (GERP++ rejected substitutions = 4.15 is	Benign (0.15)	Conflicting	Absent	NA	Het	in trans by whatsh	PM2, PP3, PP1-supporting	VUS	This study
11-119216272-TG-T	*MFRP*	NM_031433.4:c.498delC	p.Asn167ThrfsTer25	0.0001216	18.5	0.5	NA	NA	Pathogeni	DM	7	Het	in trans by familia	PVS1, PS4, PM2, PP1-strong	Pathogenic	Almoallem et al., 2020; Bacci et al., 2020; Sundin et al., 2005; Kannabiran et al., 2012; Neri et al., 2012; Ritter et al., 2013; Zacharias et al., 2015; Guo et al., 2019; Morillo Sanchez et al., 2019
11-119216596-AG-A	*MFRP*	NM_031433.4:c.313delC	p.Leu105CysfsTer32	Absent	11	0	NA	NA	Absent	Absent	NA	Het	in trans by whatsh	PVS1, PM2	Likely Pathogenic	This study
17-31267932-G-C	*TMEM98*	NM_001033504.1:c.602G>C	p.Arg201Pro	Absent	8	5	Pathogenic computational verdict because 7 pathogenic predictions from DANN, EIGEN, FATHMM-MKL, M-CAP, MutationTaster, PrimateAI and SIFT vs 4 benign predictions from DEOGEN2, MVP, MutationAssessor and REVEL.	Uncertain(0.44)	Absent	Absent	NA	Het	NA	PM2, PP3, PP1-supporting	VUS	This study
2-233386849-C-A	*PRSS56*	NM_001195129.1:c.425C>A	p.Thr142Lys	0.0000417	10	3	Pathogenic computational verdict because 4 pathogenic predictions from DEOGEN2, FATHMM-MKL, M-CAP and MutationAssessor vs 3 benign predictions from DANN, MVP and PrimateAI.	NA	Absent	Absent	NA	Hom	NA	PM2, PP3	VUS	This study
2-233387284-C-A	*PRSS56*	NM_001195129.1:c.506C>A	p.Ala169Glu	Absent	7	1	Benign computational verdict because 6 benign predictions from DANN, DEOGEN2, FATHMM-MKL, MVP, MutationAssessor and PrimateAI vs 1 pathogenic prediction from M-CAP and the position is not conserved (GERP++ rejected substitutions = 1.22 is less than 5.5).	NA	Absent	Absent	NA	Hom	NA	PM2, BP4	VUS	This study
2-233387526-G-A	*PRSS56*	NM_001195129.1:c.661G>A	p.Ala221Thr	Absent	8	1	Benign computational verdict because 6 benign predictions from DANN, DEOGEN2, FATHMM-MKL, MVP, MutationAssessor and PrimateAI vs 1 pathogenic prediction from M-CAP and the position is not conserved (GERP++ rejected substitutions = 2.42 is less than 5.5).	NA	Absent	Absent	NA	Het	NA	PM2	VUS	This study
2-233387881-G-C	*PRSS56*	NM_001195129.1:c.818G>C	p.Gly273Ala	Absent	9.5	2.5	Pathogenic computational verdict because 5 pathogenic predictions from DEOGEN2, FATHMM-MKL, M-CAP, MutationAssessor and PrimateAI vs 2 benign predictions from DANN and MVP.	NA	Absent	Absent	NA	Het	in trans by whatsh	PM2, PP3	VUS	This study
2-233387913-G-T	*PRSS56*	NM_001195129.1:c.849+1G>T	NA	0.00008335	14	2	dbscSNV ADA 0.9999, RF 0.924	splice AI Exome and Genome : Splice Altering (	Absent	Absent	NA	Het	in trans by whatsh	PVS1, PM2, PM3-Strong	Pathogenic	Siggs et al., 2020
2-233388237-G-C	*PRSS56*	NM_001195129.1:c.961G>C	p.Val321Leu	Absent	9.5	2.5	Pathogenic computational verdict because 5 pathogenic predictions from DEOGEN2, FATHMM-MKL, M-CAP, MutationAssessor and PrimateAI vs 2 benign predictions from DANN and MVP.	NA	Absent	Absent	NA	Het	in trans by whatsh	PM2, PP3	VUS	This study
2-233388527-A-AC	*PRSS56*	NM_001195129.1:c.1066dupC	p.Gln356ProfsTer152	0.0003668 (RF)	12	1	NA	NA	Pathogeni	Absent	NA	Het	in trans by whatsh	PVS1, PM2	Likely Pathogenic	Siggs et al., 2020; Almoallem et al., 2020
2-233388527-AC-A	*PRSS56*	NM_001195129.1:c.1066delC	p.Gln356ArgfsTer148	0.00005792 (RF)	17	0	NA	NA	Absent	Absent	NA	Hom	NA	PVS1, PM2	Likely Pathogenic	This study
2-233390055-C-T	*PRSS56*	NM_001195129.1:c.1651C>T	p.Leu551Phe	0.000007895	5.5	1.5	Benign computational verdict because 5 benign predictions from DANN, DEOGEN2, MVP, MutationAssessor and PrimateAI vs 2 pathogenic predictions from FATHMM-MKL and M-CAP and the position is not conserved (GERP++ rejected substitutions = 4.07 is less than 5.5).	NA	Absent	Absent	NA	Het	NA	PM2	VUS	This study

VUS, variant of unknown significance; HGVSc, human genetic variant source cDNA; HGVSp, human genetic variant source protein; gnomAD AF, allele frequency in gnomAD database

Table S2: Clinical features of nanophthalmos and high hyperopia families carrying MFRP, PRSS56, TMEM98 deleterious variants

Patient #	Family #	Source	Age at exam	Sex	Ethnicity	Alleles	Gene	Sporadic/ Familial	logMAR VA		Phakic Refraction (SE)		Refraction Type	Lens status		Axial length (mm)		Phakic ACD (mm)		C/D Ratio		Max Clinic IOP		Scleral Thickness (mm)		Glaucoma	Narrow angle?	Macular phenotype	Choroidal Folds	Pigmentary retinopathy	Drusen	Vascular Tortuosity	Strabismus	Other findings
									OD	OS	OD	OS		OD	OS	OD	OS	OD	OS	OD	OS	OD	OS	OD	OS									
P01131	F1	UM	64	F	MIXD	2	*MFRP*	Familial	1.0	CF 1'	12.25	12.25	Manifest	Phakic	Phakic	15.8	15.9	NR	NR	No view	1	34	16	ND	ND	+	NR	NR	NR	NR	NR	NR	NR	-
P01595	F1	UM	45	M	MIXD	2	*MFRP*	Familial	ND	ND	16.13	15.88	Wearing	Phakic	Phakic	15.5	16.1	NR	NR	NR	NR	NR	NR	ND	ND	NR	NR	NR	NR	NR	NR	NR	NR	-
P01600	F1	UM	64	F	MIXD	2	*MFRP*	Familial	bare HM	0.7	12.50	11.88	Wearing	Pseudo	Pseudo	16.9	18.9	2.13	NR	0.6	NR	28	19	2.3	1.8	+	+	-	+ effusion	+	-	+	-	Guttata
P01604	F1	UM	68	M	MIXD	2	*MFRP*	Familial	0.9	0.9	15.75	16.13	Wearing	Pseudo	Pseudo	15.5	15.6	1.13	1.63	NR	NR	12	18	2.3	2.4	+	+	ERM	+ effusion	+	-	+	-	CRVO
P01605	F1	UM	77	M	MIXD	2	*MFRP*	Familial	0.7	1.0	18.50	18.50	Wearing	Pseudo	Pseudo	16.0	16.5	2.44	2.21	0.3	0.3	24	19	2.6	2.7	+	+	-	-	NR	NR	+	-	-
P02210	F2	UM	56	M	EUWA	2	*MFRP*	Sporadic	1.2	1.2	19.38	24.25	Manifest	Phakic	Phakic	13.7	13.6	NR	NR	NR	NR	10	10	2.5	2.3	-	NR	CME	+	+	-	+	-	-
P05188	F3	UM	61	M	EUWA	2	*MFRP*	Sporadic	HM	0.8	11.75	12.25	NR	Pseudo	Pseudo	17.7	17.2	NR	2.29	pallor	0.2	34	16	2.1	1.8	-	+	-	+	+	-	-	ET	-
P05208	F4	UM	18	F	EUWA	2	*MFRP*	Familial	0.5	0.5	17.25	18.25	Wearing	Phakic	Phakic	14.5	14.4	NR	NR	0.15	0.15	18	13	2.4	2.5	-	+	Retinal folds	-	NR	+	+	ET	-
P05209	F4	UM	14	M	EUWA	2	*MFRP*	Familial	0.1	0.0	15.63	15.75	Wearing	Phakic	Phakic	16.3	16.1	NR	NR	0.4	0.3	18	13	ND	ND	-	+	Retinal folds	-	NR	+	+	-	-
D1108-001	F5	NEI	32	F	EUWA	2	*MFRP*	Sporadic	0.4	0.3	8.63	8.38	Manifest	Pseudo	Pseudo	19.8	19.7	ND	ND	0	0	16	18	ND	ND	-	NR	CME	-	+	-		-	optic disc edema
P04556	F6	UM	24	M	EUWA	1	*MFRP*	Sporadic	NR	NR	11.13	11.50	Wearing	NR	NR	17.2	17.1	NR	NR	0.5	0.3	23	28	ND	ND	+	NR	Retinal folds	+	-	+	NR	NR	-
P05050	F7	UM	59	F	EUWA	1	*MFRP*	Sporadic	0	0.0969	1.25	1.88	Wearing	Pseudo	Pseudo	19.9	19.3	2.32	3.82	0.2	0.1	15	17	2.4	ND	+	+	CME, ERM	-	-	+	+	-	aqueous misdirection
P02228	F9	UM	55	M	EUWA	2	*PRSS56*	Familial	0.6	0.6	10.75	9.38	Wearing	Phakic	Phakic	NR	17.5	NR	NR	NR	0.8	38	36	+thick	+thick	+	NR	NR	+ effusion	NR	NR	NR	NR	aqueous misdirection
P02239	F9	UM	70	F	EUWA	2	*PRSS56*	Familial	1.0	0.5	13.50	13.50	Wearing	Phakic	Phakic	17.1	17.4	2.62	2.62	0.1	0.1	18	18	2.1	+thick	-	+	-	+	NR	-	-	-	ptosis
P02302	F9	UM	65	F	MIXD	2	*PRSS56*	Familial	1.0	0.4	10.50	11.00	Wearing	Pseudo	Pseudo	17.5	17.5	NR	NR	NR	NR	23	12	+thick	+thick	+	+	-	+	+	-	-	-	ptosis
MISC0005-1	F10	NEI	46	M	EUWA	2	*PRSS56*	Familial	0.3	0.5	17.75	16.75	Manifest	Phakic	Phakic	15.4	15.2	NR	NR	0.2	0.2	16	16	ND	ND	-	+	ND	ND	-	ND	-	-	
MISC0005-2	F10	NEI	40	F	EUWA	2	*PRSS56*	Familial	0.5	0.5	14.63	14.13	Manifest	Phakic	Phakic	16.7	16.6	NR	NR	0.1	0	15	15	ND	ND	-	+	-	+	-	+	+	-	
MISC0005-3	F10	NEI	16	F	EUWA	2	*PRSS56*	Familial	0.4	CF 6"	13.00	13.63	wearing	Phakic	Phakic	15.7	15.1	NR	NR	0	*0*	16	16	ND	ND	-	+	ERM	ND	-	+	-	ET	
P04927	F11	UM	61	M	EUWA	2	*PRSS56*	Familial	0.5	NLP	25.00	25.00	Wearing	Aphakic	Aphakic	15.7	16.3	NR	NR	0.1	NR	15	16	+thick	+thick	+	+	Retinoschesis	-	+	+	-	-	retinal detachment, ptosis
P02206	F12	UM	75	M	EUWA	2	*PRSS56*	Sporadic	0.3	0.3	15.00	14.25	Wearing	Phakic	Phakic	16.0	15.8	NR	2.07	0.7	0.95	30	34	2	+thick	+	+	Retinoschesis	+ effusion	+	+	+	ET	aqueous misdirection
P05214	F12	UM	61	F	EUWA	1	*PRSS56*	Sporadic	1.1	0.1	NR	NR	NR	Pseudo	Pseudo	19.6	19.9	1.4	2.53	0.15	NR	68	10	2.9	2.9	+	+	-	+ effusion	-	-	-	XT	aqueous misdirection, ptosis
P02367	F13	UM	85	M	EUWA	1	*PRSS56*	Sporadic	1.0	0.2	2.88	2.25	Manifest	Pseudo	Pseudo	20.6	20.9	NR	NR	0.95	0.75	42	20	+thick	+thick	+	+	NR	NR	NR	NR	+	-	CRVO, NVG
P01811	F14	UM	60	F	EUWA	1	*TMEM98*	Familial	0.3	0.6	NR	NR	NR	Pseudo	Pseudo	17.3	17.3	NR	2.13	0.85	0.2	35	NR	+thick	+thick	+	+	-	+ effusion	NR	NR	+	-	
P01963	F14	UM	30	F	EUWA	1	*TMEM98*	Familial	0.1	0.1	9.8	10.3	Wearing	Phakic	Phakic	NR	NR	NR	NR	0.2	0.2	16	16	ND	ND	NR	NR	NR	NR	NR	NR	NR	NR	NR
P02232	F14	UM	57	F	EUWA	1	*TMEM98*	Familial	0.6	0.4	10.6	10.0	Wearing	Pseudo	Pseudo	NR	NR	NR	NR	0.95	0.95	35.8	35.8	ND	ND	+	+	NR	NR	NR	NR	NR	NR	NR
P02233	F14	UM	84	F	EUWA	1	*TMEM98*	Familial	CF	0.6	8.9	8.9	Wearing	Pseudo	Pseudo	NR	NR	NR	NR	0.3	0.4	13	10	ND	ND	NR	NR	NR	NR	NR	NR	NR	NR	NR

SE, spherical equivalent, ACD, anterior chamber depth, C/D cup-to-disc; IOP, intraocular pressure; logMAR VA, log minimal angle of resolution visual acuity; OD, right; OS, left; NR, not recorded, ND, not determined; EUWA, European white; MIXD, mixed race; NEI, National Eye Institute; UM, University of Michigan; ERM, epiretinal membrane; CME, cystoid macular edema; ET, esotropia; XT, exotropia; CRVO, central retinal vein occlusion; NVG, neovascular glaucoma
